# Unlocking the Potential of Ultra-High Dose Fractionated Radiation for Effective Treatment of Glioblastoma in Mice

**DOI:** 10.7150/jca.95148

**Published:** 2024-05-30

**Authors:** Xiao-Yan Lan, Lukasz Kalkowski, Cheng-Yan Chu, Anna Jablonska, Shen Li, Mihoko Kai, Yue Gao, Miroslaw Janowski, Piotr Walczak

**Affiliations:** 1 Department of Neurology, Dalian Municipal Central Hospital, Dalian, China.; 2 Department of Diagnostic Radiology and Nuclear Medicine, University of Maryland Baltimore, Baltimore, MD, USA.; 3 Department of Neurology and Psychiatry, Beijing Shijitan Hospital, Capital Medical University, Beijing, China.; 4 Department of Radiation Oncology, Johns Hopkins University, School of Medicine, Baltimore, MD, USA.

**Keywords:** glioblastoma, fractionated radiation, brain injury, vascular damage, hypoxia-inducible factor

## Abstract

**Background:** Current radiotherapy regimens for glioblastoma (GBM) have limited efficacy and fails to eradicate tumors. Regenerative medicine brings hope for repairing damaged tissue, opening opportunities for elevating the maximum acceptable radiation dose. In this study, we explored the effect of ultra-high dose fractionated radiation on tumor responses and brain injury in immunocompetent mice which can better mimic the tumor-host interactions observed in patients. We also evaluated the role of the hypoxia-inducible factor-1 alpha under radiation as potential target for combating radiation-induced brain injury.

**Methods**: Naïve and Hif-1α^+/-^ heterozygous mice received a fractionated daily dose of 20 Gy for three or five consecutive days. Magnetic resonance imaging (MRI) and histology were performed to assess brain injury post-radiation. The 2×10^5^ human GBM1 luciferase-expressing cells were transplanted with tolerance induction protocol. Fractionated radiotherapy was performed during the exponential phase of tumor growth. Bioluminescence imaging, MRI, and immunohistochemistry staining were performed to evaluate tumor growth dynamics and radiotherapy responses. Additionally, animal lifespan was recorded.

**Results**: Fractionated radiation of 5×20 Gy induced severe brain damage, starting 3 weeks after radiation. All animals from this group died within 12 weeks. In contrast, later onset and less severe brain injury were observed starting 12 weeks after radiation of 3×20 Gy. It resulted in complete GBM eradication and survival of all treated animals. Furthermore, Hif-1α^+/-^ mice exhibited more severe vascular damage after fractionated radiation of 3×20 Gy.

**Conclusion**: Ultra-high dose fractionated 3×20 Gy radiation has the potential to fully eradicate GBM cells at the cost of only mild brain injury. The Hif-1α gene is a promising target for ameliorating vascular impairment post-radiation, encouraging the implementation of neurorestorative strategies.

## Introduction

Glioblastoma (GBM) is the most common primary central nervous system malignancy in adults 1; the three-year survival rate of patients is only 10.3% 2. This dismal clinical outcome highlights that treating GBM remains one of the greatest challenges in medicine.

Radiotherapy is an essential adjuvant strategy used in conjunction with other treatments 3. However, the negative impact of side effects, particularly on neurological and cognitive function 4, can significantly reduce the quality of life for patients after treatment. Furthermore, these side effects often set the threshold for the maximum tolerable levels of anti-tumor radiation therapies that can be administered. Several targeted therapeutic techniques have been developed to address these issues, including three-dimensional conformal radiation therapy 5, intensity-modulated radiation therapy 6, functional imaging-guided dose-escalated radiation therapy 7 and ultra-high-dose-rate radiotherapy (FLASH) 8, 9. These techniques can selectively destroy the bulk of the tumor. Unfortunately, the early invasive growth of GBM, with satellite cancer cells found within the brain far from the tumor mass, makes conformal radiation therapy prone to a high recurrence rate. As a result, there is a dire need for a breakthrough treatment.

The field of regenerative medicine has advanced tremendously in recent years, paralleling the progress in oncology. However, these fields have not yet been sufficiently integrated, leaving many untapped opportunities. Regenerative medicine techniques now enable the repair and replacement of damaged white matter and vasculature, the tissue components most vulnerable to radiation damage 10. It presents an exciting opportunity for elevating the maximum acceptable dose of radiation. Combining ultra-high dose fractionated radiation therapy with brain repair techniques may be feasible to achieve effective brain tumor eradication. In addition, we sought to explore the mechanism of radiation-induced vascular damage. To do so, we studied hypoxia-inducible factor-1 alpha (Hif-1α) signaling, a well-established system that is a master regulator of endothelial homeostasis and angiogenesis 11. Hif-1α signaling has not been systematically studied in the context of responses to radiation injury, and the modulation of Hif-1α signaling has not been exploited as a therapeutic strategy for brain radiation injury. In this study, we explored the efficiency of ultra-high dose fractionated radiation therapy for human GBM in immunocompetent mice using the described immunological tolerance induction protocol 12. We also investigated radiation-induced brain injury in both wild-type mice and Hif-1α^+/-^ heterozygote mice to better understand the role of Hif-1α signaling in radiation-induced vascular damage.

## Materials and Methods

### Animal irradiation

All procedures involving live animals were approved and performed in accordance with the Guide for the Care and Use of Laboratory Animals at the local Animal Care and Use Committee. Ten naïve C57BL/6J mice (8-10 weeks (w), 20-25 g, Jackson Laboratory), as well as 12 transgenic Hif-1α^+/-^ mice (which are heterozygous for a null (knockout) allele at the locus encoding Hif-1α) and 12 wild-type mice of the same litter (6-8 w, both provided by Prof. Gregg L. Semenza), received fractionated daily dose of 20 Gy radiation for consecutive three (Fr 3×20 Gy) or five (Fr 5×20 Gy) days. Briefly, the mice were anesthetized with isoflurane (4% for induction, 1%-2% for maintenance) and placed in the prone position. A custom-built small animal radiation research platform equipped with onboard computed tomography (CT) guidance was used for radiotherapy. Following three-dimensional CT acquisition, a target spot (2.5 mm deep relative to the skull) was irradiated with a single beam (60° angle), using a 5×5 mm^2^ collimator at a daily dose of 20 Gy for consecutive three or five days.

### Magnetic resonance imaging

Magnetic resonance imaging (MRI) was performed under general anesthesia (isoflurane) after radiation 1 w, 3 ws, 5 ws, and then monthly until the mice deteriorated or reached the end of our observation time. Mice were placed on a water-heated animal bed equipped with temperature and respiratory control. All MRI experiments were performed on a horizontal bore 11.7 T Bruker Biospec system (Bruker, Ettlingen, Germany), and a surface coil array was used for image acquisition. Baseline T2 (repetition time (TR)/echo time (TE)=2500/30 ms, slice thickness=0.7 mm, average (AV)=2, the field of view=14×14 mm^2^, matrix size=256×256, RARE factor=8)-weighted and T1 (TR/TE 350/6.7 ms, AV=2)-weighted images of the brain were acquired. Gadolinium (30 μL) was injected intraperitoneally (i.p.) for contrast-enhanced T1 scans, and T1 post-gadolinium images were acquired.

### Cell culture

The human GBM1 luciferase-expressing cells were kindly provided by Dr. Charles Eberhart from Johns Hopkins University. Cells were expanded in culture medium (Neurobasal-A Medium and DMEM/F12 (1:1), Thermo Fisher Scientific) containing B27 without vitamin A (Thermo Fisher Scientific), 20 ng/mL human EGF (Peprotech), 20 ng/mL human bFGF (Peprotech) and 2 μg/mL heparin (Sigma). All cells were suspension cultured as neurospheres in a humidified atmosphere of 5% CO_2_ at 37 °C.

### Tumor implantation

Fifteen male C57BL/6J mice (6-8 w, 20-25 g, Jackson Laboratory) were used for tumor transplantation. Animals were randomly assigned to three groups: GBM1 without radiation (GBM1, n=5), GBM1 with a fractionated daily dose of 2 Gy (GBM1+Fr 3×2 Gy, n=5) or 20 Gy (GBM1+Fr 3×20 Gy, n=5) for consecutive three days. Before transplantation, GBM1 oncospheres were harvested, dissociated into single cells, and suspended in PBS at a final concentration of 1×10^5^/μL. Then animals were anesthetized with isoflurane. A total number of 2×10^5^ cells were injected at a rate of 1 μL/min into the right striatum (anteroposterior=0.5 mm; mediolateral=2.0 mm; dorsoventral=2.5 mm) using a 10 μL Hamilton syringe with an attached 31-gauge needle. After injection, the needle was kept in place for 2 minutes (min) to avoid backflow of the injected cells through the needle tract and then withdrawn.

To induce immunological tolerance to xenografted human tumors in immunocompetent C57BL/6J mice, hamster anti-mouse CD154mAb (MR1, BioXcell, Lebanon, NH) and CTLA-4-Ig (Abatacept, Bristol-Myers Squibb, Princeton, NJ) were administered to animals (500 μg each) i.p. on days 0, 2, 4, and 6 after tumor inoculation. The weight changes and neurological symptoms of mice were recorded twice a week. Bioluminescence imaging (BLI) and MRI were used to confirm the successful establishment of tumor implantation and monitor tumor growth. Fractionated radiotherapy (Fr 3×2 Gy or Fr 3×20 Gy) was performed after tumor inoculation when BLI showed accelerating tumor growth. Then BLI and MRI were performed to assess the tumor response and brain injury.

### Bioluminescence imaging

Animals were anesthetized with isoflurane and injected i.p. with 150 μL luciferin (30 mg/mL, Gold Biotechnology). Images were acquired 5-15 min after substrate injection at the peak of the bioluminescence signal by IVIS Spectrum In Vivo Imaging System (PerkinElmer). For BLI analysis, images were quantified by drawing regions of interest (ROIs). The data were expressed as photon flux (p/sec). Imaging began on day 1 post-implantation and was then done weekly within the first month, biweekly within the second month, and monthly after that until the mice deteriorated or reached the end of our observation time.

### Behavior assessment

Open-field test: Open-field test was performed using the SDI Open Field System (San Diego Instruments, San Diego, CA). The open field arena was a 20×40 cm Lexan shoebox cage equipped with a camera to monitor overall locomotor activity. For each open-field test session, mice were placed in the center of the field, and activity was monitored for 5 min. In addition, the travel distance and average speed were measured by the investigator blind to the study design.

Y-maze test: Mice were placed at the end of one of the three arms and allowed to explore freely for 5 min. Spatial recognition memory was assessed. The testing was video recorded, and the number of times the mouse entered three different arms consecutively divided by the total visits were measured by an observer blind to the condition.

### Histological analysis

Mice were deeply anesthetized with isoflurane and perfused intracardially with 5% sucrose, followed by 4% paraformaldehyde. Brains were dissected, post-fixed in 4% paraformaldehyde overnight at 4 ℃, then successively cryopreserved in 20% and 30% sucrose until the tissue sank. Brains were cryosectioned into 30-μm-thick coronal sections. Hematoxylin and eosin (H&E) staining was performed. For eriochrome cyanin staining, slides were oven-dried and dehydrated in 95% and 70% ethanol, then put into the eriochrome cyanine solution (0.2% eriochrome cyanine, 0.4% FeCl_3,_ and 0.5% H_2_SO_4_). Staining was developed by alternating exposure to 0.1% NH_4_OH for 3-7 s and rinsing in distilled water for 30 s until the blue background was reduced, and the cells turned faintly pink but still had blue shading. After that, sections were put in two changes of 70%, 95%, and 100% ethanol and three changes of xylene for 10 min each.

For immunofluorescence staining, the sections were blocked using 0.1% Triton and 2% bovine serum albumin for 1 h at room temperature and then incubated overnight with primary antibodies at 4 ℃. In addition, either Alexa-488 or Alexa-555 (Molecular Probes, 1:200) secondary antibody was added for 2 h incubation at room temperature. Sections were then counterstained with mounting medium with DAPI (VectaStain, Vector Labs). The following primary antibodies were used: human-nuclear antigen (HuNu, 1:250; Cat. MAB1281, Millipore), Stem 121 (1:500; Cat. Y40410, Takara), GFAP (1:250; Cat. Z0334, Dako), NeuN (1:100; Cat. D3S3I, Cell Signaling Technology), Iba1 (1:250; Cat. 019-19741, Wako), CD45 (1:250; Cat. ab10558, Abcam), CD68 (1:300; Cat. ab125212, Abcam), Collagen IV (1:300; Cat. ab6586, Abcam). Histochemical and immunofluorescent images were acquired with a DMi8 inverted microscope (Leica Microsystems). For quantification, images of entire brain tissue (three to five brain tissue sections per mouse) were captured with 5× magnification. In addition, the intensities of the ipsilateral or contralateral hemisphere were measured by Image J (Version 1.52p, USA).

### Statistical analysis

The overall mouse survival time was calculated and expressed by the Kaplan-Meier curve, and the statistical difference was analyzed by log-rank tests. The Shapiro-Wilk test was used to test if the distribution was normal. Those results were presented as mean ± standard deviation. The intergroup differences between more than 2 groups were assessed using ANOVA followed by a post-hoc the least significance difference test. The comparison of 2 groups was evaluated using student t-test or paired t-test. Data without normal distribution were presented as median with interquartile range. The Mann-Whitney test was used when comparing two groups, and the Kruskal-Wallis test was used when comparing more than 2 groups. All statistical analysis was performed using the SPSS software package (Version 22.0, USA). P<0.05 was considered to be statistically significant.

## Results

### Ultra-high dose fractionated radiation leads to late-phase brain injury

**Figure 1:**
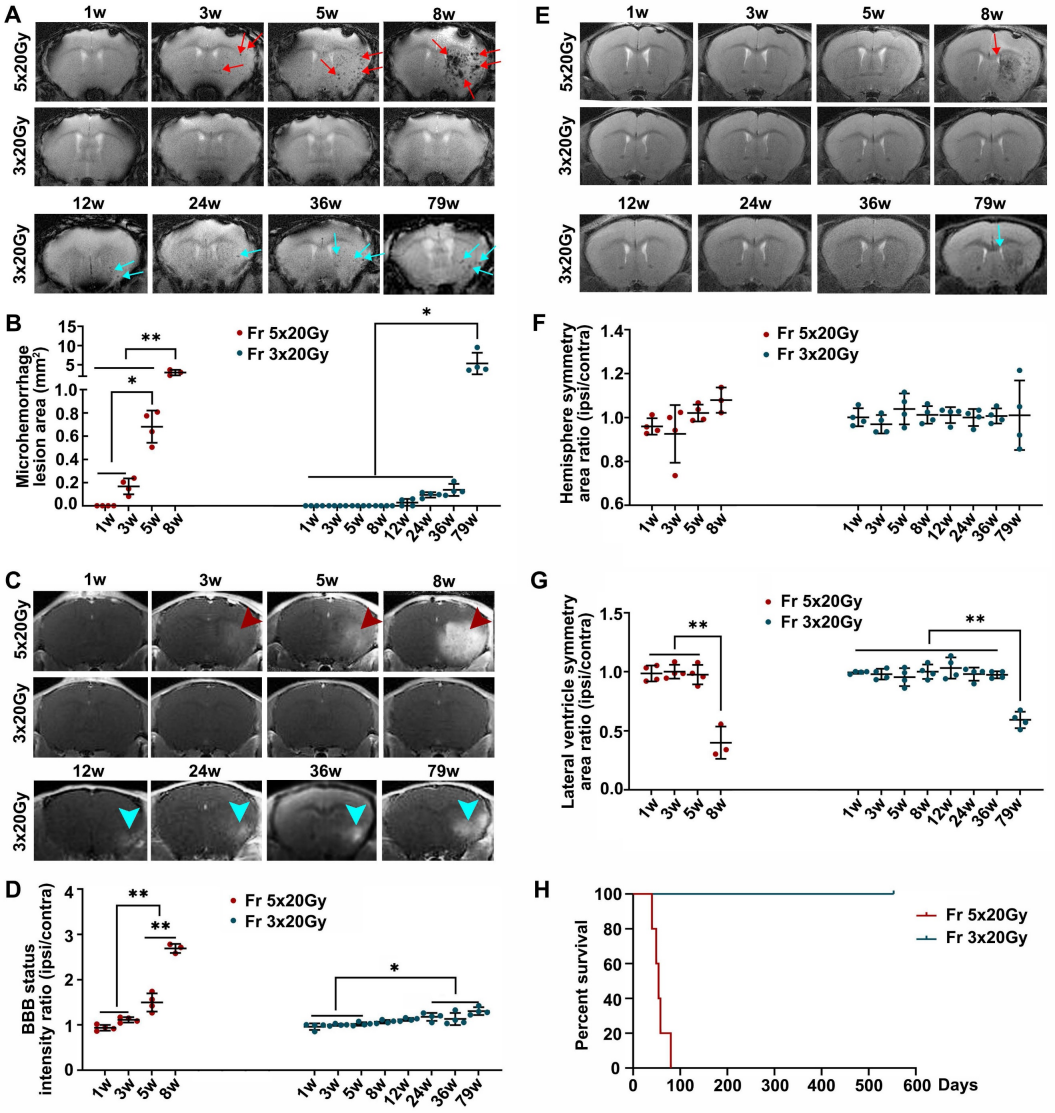
** Ultra-high dose fractionated radiation leads to late-phase brain injury.** (A) Representative T2* images showed the microhemorrhages in mice receiving ultra-high fractionated 5×20 Gy (Fr 5×20 Gy) radiation starting from 3 weeks (red arrow) and 3×20 Gy (Fr 3×20 Gy) radiation starting from 12 weeks (blue arrow) after radiation. (B) Quantitative analysis of microhemorrhage lesion area, which was calculated by summing the areas of all microbleeding lesions in each mouse. (C) Representative T1-weighted gadolinium-enhanced MR images showed the BBB breakdown in mice receiving Fr 5×20 Gy radiation (hyperintensity, red arrowhead) and Fr 3×20 Gy radiation (blue arrowhead). (D) Quantification analysis of BBB breakdown by intensity ratio (ipsilateral/contralateral) for each mouse. (E) Representative T2-weighted MR images showed the anatomical structures of mice receiving fractionated radiation. Lateral ventricle compression was observed at 8 weeks after Fr 5×20 Gy radiation (red arrow) and at 79 weeks after Fr 3×20 Gy radiation (blue arrow). (F) Quantitative analysis of hemisphere symmetry area ratio (ipsilateral/contralateral) after radiation. (G) Quantitative analysis of lateral ventricle symmetry area ratio (ipsilateral/contralateral) for mice receiving fractionated radiation. ANOVA. N=4 mice/group. ^*^P<0.05, ^**^P<0.01. (H) Kaplan-Meier curves of mice receiving radiations showed that all mice from Fr 5×20 Gy group died within 12 weeks, while mice from the Fr 3×20 Gy group survived to the end of our observation time (80 weeks) post-irradiation. N=5 mice/group.

We studied the effects of ultra-high dose radiation on the brain of immunocompetent mice. The mice were exposed to two radiation doses, 60 Gy divided into 3 fractions (Fr 3×20 Gy) and 100 Gy divided into 5 fractions (Fr 5×20 Gy) delivered daily. Longitudinal MRI showed that Fr 5×20 Gy radiation-induced multiple microhemorrhages starting from 3 weeks after radiation (Figure 1A-B) and that was accompanied by blood-brain barrier (BBB) breakdown (Figure 1C-D). Additionally, we observed white matter damage and brain edema as evidenced by the ipsilateral lateral ventricle compression 8 weeks after Fr 5×20 Gy radiation (Figure 1E-G). Most of the Fr 5×20 Gy animals showed sudden deterioration, and all mice died within 12 weeks (Figure 1H).

**Figure 2:**
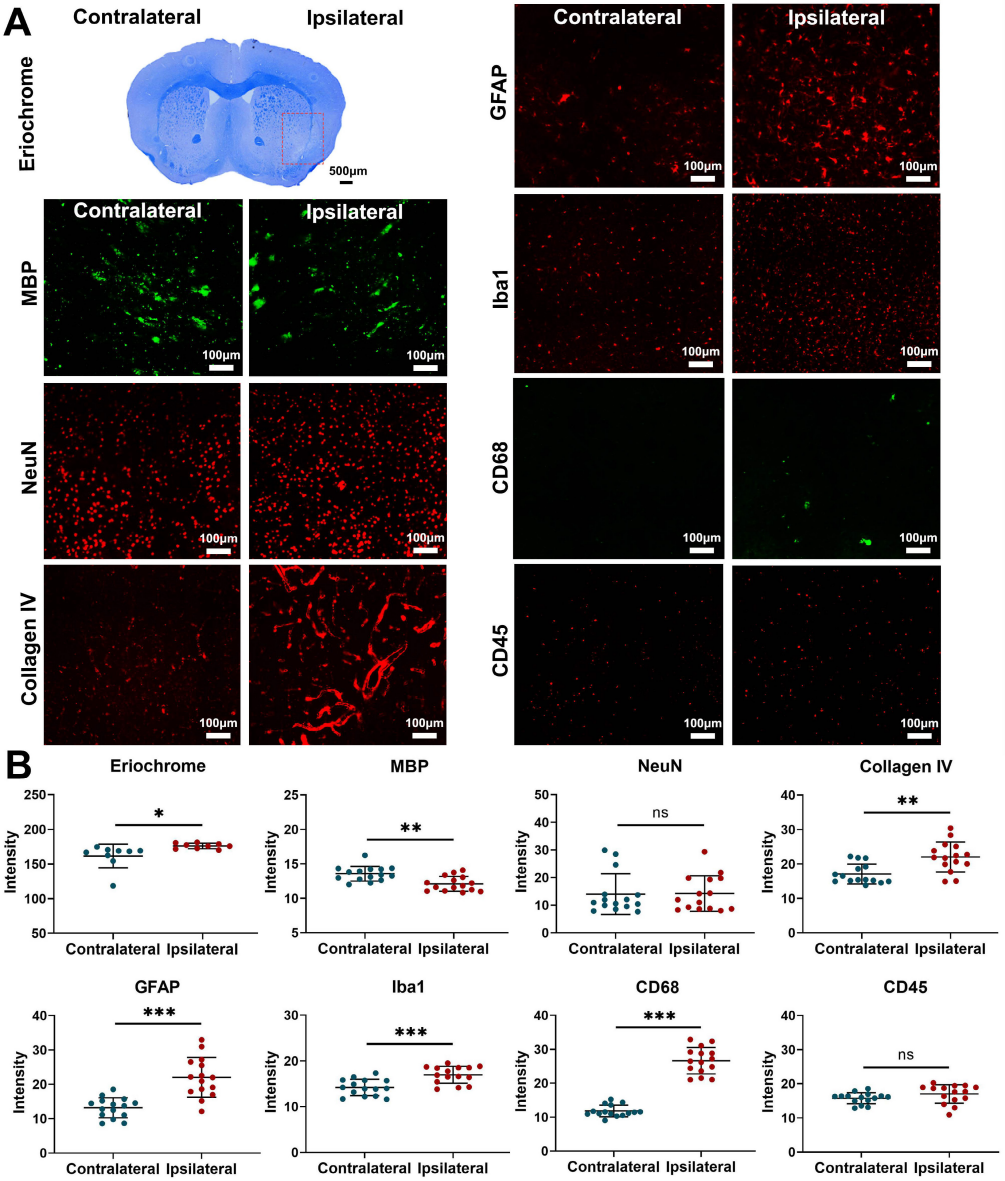
** Fractionated 3×20 Gy radiation causes mild demyelination and vascular abnormalities, without affecting neurons.** (A) Representative histology/ immunohistochemistry of brain tissue stained with Eriochrome (myelin), MBP (a marker of myelin), NeuN (a marker of neuron), Collagen IV (a marker of blood vessel), GFAP (a marker of astrocyte), Iba1 (a marker of microglia), CD68 (a marker of macrophage), and CD45 (a marker of leukocyte) at 80 weeks after fractionated 3×20 Gy radiation. The scale bars are 500 μm for Eriochrome image and 100 μm for other high-power images. (B) Quantification of signal intensity in the contralateral and ipsilateral hemispheres for each staining. Paired t-test. N=3 ROIs from 3 (Eriochrome staining) or 5 (all the immunofluorescence staining) brain slices in each group. ^*^P<0.05, ^**^P<0.01, ^***^P<0.001. ns: no significant difference.

Fractionated radiation of 3×20 Gy also caused brain damage, but the damage was observed much later, after three months. Animals developed vascular abnormalities manifested as microbleeds and BBB breakdown, first observed 12 weeks after radiation (Figure 1A-D). At 79 weeks after radiation, we observed compression of the lateral ventricle on the ipsilateral side (Figure 1E-G). We conducted histological staining in the very late stage (80 weeks) after radiation to further assess brain damage. As shown in Figure 2, only mild demyelination (Eriochrome, MBP) and vascular abnormalities (Collagen IV) were observed. Minor neuroinflammation, including astrocyte (GFAP) or microglia (Iba1)/macrophage (CD68) activation, was detected at 80 weeks. However, we found no neuronal loss (NeuN) even at that late time. All mice from the Fr 3×20 Gy group survived as long as 80 weeks (the end of our observation time) post-irradiation. Therefore, this dose was selected for further GBM1 eradication studies. The remarkably long window encourages further study to identify targets and opportunities to implement therapeutic intervention addressing the observed damage.

### Fractionated 3×20 Gy radiotherapy eradicated GBM in immunocompetent mice

**Figure 3:**
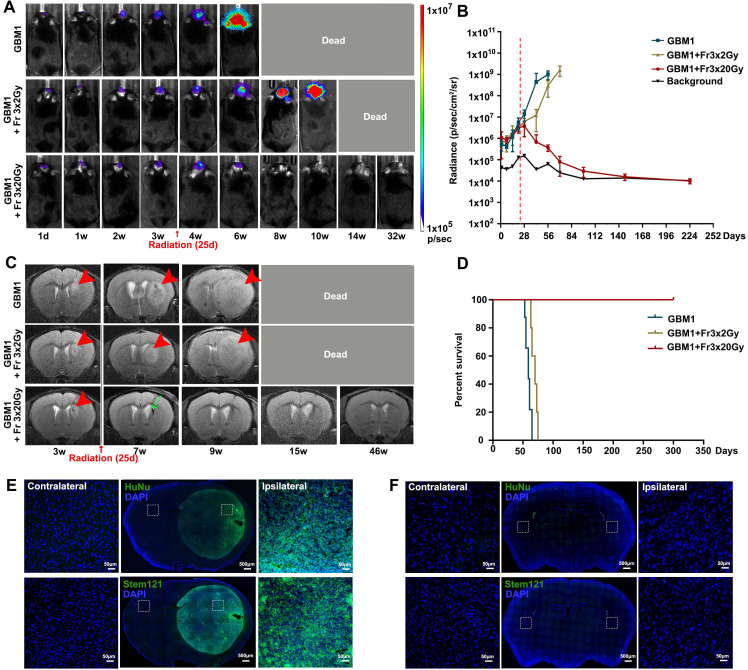
** Ultra-high dose fractionated 3×20 Gy radiotherapy successfully eradicates the GBM1 tumor**. (A) Representative BLI images of non-irradiation (GBM1), fractionated 3×2 Gy (GBM1+Fr 3×2 Gy, a low-dose control) or 3×20 Gy (GBM1+Fr 3×20 Gy) irradiation-treated tumor-bearing mice before and after radiation at the indicated days. Irradiation was started on days 25 after tumor inoculation. The number of human GBM1 cells was represented by the defined color shade. (B) Quantification of the BLI signal revealed that fractionated 3×20 Gy radiation completely eradicates the GBM1 tumor cells. The dot-dashed line represents the date of starting radiation. (C) Representative T2-weighted MR images for GBM1 mice showed a visible tumor mass around 3 weeks after tumor inoculation (red arrowhead). Mice without irradiation or with fractionated 3×2 Gy died due to the severe tumor burden. Mice with Fr 3×20 Gy radiation showed regression of cancer with some hypointensity at the tumor site (green arrow). (D) Survival curves of GBM1 mice with or without radiation. N=5 mice/group. (E) Identification of implanted xenografts by HuNu (a marker of human cell nucleus) and Stem121 (a marker of human cell cytoplasm) showed the successful implantation of human GBM1 tumor in immunocompetent mice. (F) Immunofluorescence staining of HuNu and Stem121 at 42 weeks after fractionated 3×20 Gy radiation confirmed the disappearance of the GBM1 cells. The scale bars for low-power images are 500 μm and high-power images are 50 μm.

The GBM1 tumor xenograft tolerance model was successfully established in the brain of immunocompetent recipients, and its growth was monitored with BLI and MRI. Tumors grew rapidly, as shown by both imaging modalities (Figure 3). Radiotherapy (Fr 3×2 Gy (a lower dose used as clinically relevant therapy control) or Fr 3×20 Gy) was performed when BLI showed exponential tumor growth and T2 MRI showed a visible tumor mass around 25 days after tumor inoculation. All the untreated tumor-bearing mice died within 9 weeks. A lower dose of Fr 3×2 Gy irradiation slowed the tumor growth and slightly prolonged animal survival; however, all mice died within 14 weeks. In animals treated with ultra-high dose fractionated 3×20 Gy radiation, the bioluminescence signal of the tumor gradually disappeared and overlapped with background radiance (Figure 3A-B). MR images one month later showed regression of cancer with some hypointensity at the tumor site (Figure 3C). No tumor regrowth was observed even at 46 weeks after radiation, as evidenced by MRI (Figure 3C), and there was a dramatic improvement in survival for up to 322 days (the end of our observation time, Figure 3D). Immunofluorescence staining of HuNu (a marker of human cell nucleus) and Stem121 (a marker of human cell cytoplasm) confirmed the successful implantation of human GBM1 tumor in immunocompetent mice (Figure 3E). While negative of HuNu and Stem 121 staining at 42 weeks after fractionated 3×20 Gy radiation further revealed the eradication of the GBM1 cells (Figure 3F).

### Hif-1α deficiency exacerbates local vascular damage after radiation

We further evaluated the role of the Hif-1α gene in radiation-induced brain injury. Fractionated 5×20 Gy radiation-induced severe brain injuries in Hif-1α^+/-^ heterozygote and wild-type mice. In T2*, hypointensity appeared starting at week 3 after irradiation (Supplementary Figure 1 A-B), indicating microhemorrhages. In addition, there was widespread BBB breakdown starting 3 weeks after radiation, as evidenced by Gd enhancement on T1 images (Supplementary Figure 1 C-D). The ipsilateral/contralateral hemisphere and lateral ventricle areas were measured on T2 images, which showed a volume increase at 8 weeks, probably due to hemorrhage and edema, and then reduced at 12 weeks, indicating brain atrophy (Supplementary Figure 1 E-F). However, we did not observe a significant difference in the severity of the above brain damage measures between Hif-1α^+/-^ and wild-type mice under this dose treatment.

**Figure 4:**
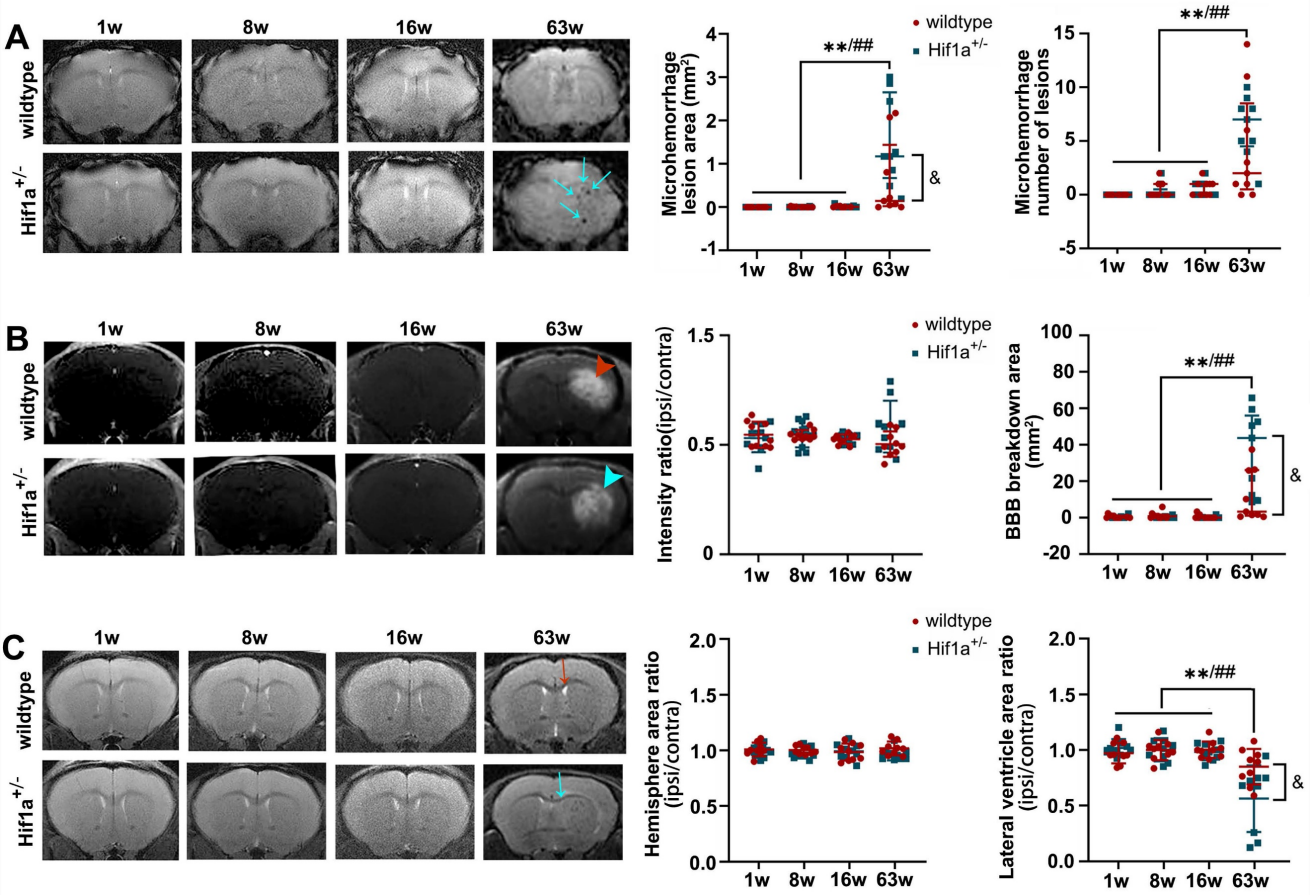
** Fractionated 3×20 Gy irradiation-induced brain damage in both Hif-1α^+/-^ heterozygote and wild-type mice**. (A) Representative T2* images and microhemorrhage assessment for Hif-1α^+/-^ heterozygote and wild-type mice receiving fractionated 3×20 Gy radiation. More microhemorrhage lesions were observed in Hif-1α^+/-^ heterozygote mice (blue arrow). (B) Representative T1-weighted gadolinium-enhanced MRI and BBB status for mice receiving fractionated radiation. Fractionated 3×20 Gy radiation-induced BBB breakdown at 63 weeks after radiation (hyperintensity, arrowhead) and more extensive BBB breakdown was observed in Hif-1α^+/-^ heterozygote mice. (C) Representative T2-weighted MR images and anatomical analysis for mice receiving fractionated radiation. Ipsilateral lateral ventricle compression was observed at 63 weeks after radiation in wildtype mice (red arrow) and Hif-1α^+/-^ mice (blue arrow), with more severe in Hif-1α^+/-^ heterozygote mice. N=3 mice per group (3 slices for each mouse). Wildtype group: ANOVA, ^*/**^P<0.05/0.01. Hif-1α^+/-^ group: ANOVA, ^#/##^P<0.05/0.01. Wildtype vs. Hif-1α^+/-^ group: Student t-test, ^&^P<0.05.

There was no apparent brain injury before 16 weeks post-fractionated 3×20 Gy irradiation. However, animals developed vascular abnormalities manifested as microbleeds and BBB breakdown at 63 weeks after radiation in both Hif-1α^+/-^ heterozygote and wild-type mice (Figure 4A-B). In addition, at 63 weeks, we observed compression on the ipsilateral side, indicating microhemorrhages and/or brain edema (Figure 4C). Neither neurobehavioral manifestation (open-field and Y-maze tests) nor survival time showed the difference between Hif-1α^+/-^ heterozygote mice and wild-type mice under any radiation dose (Supplementary Figure 2). Yet it is worth noting that, compared with wild-type mice, there was a clear trend towards more microhemorrhages, more extensive BBB breakdown, and more ipsilateral lateral ventricle compression in Hif-1α^+/-^ heterozygote mice (Figure 4).

**Figure 5:**
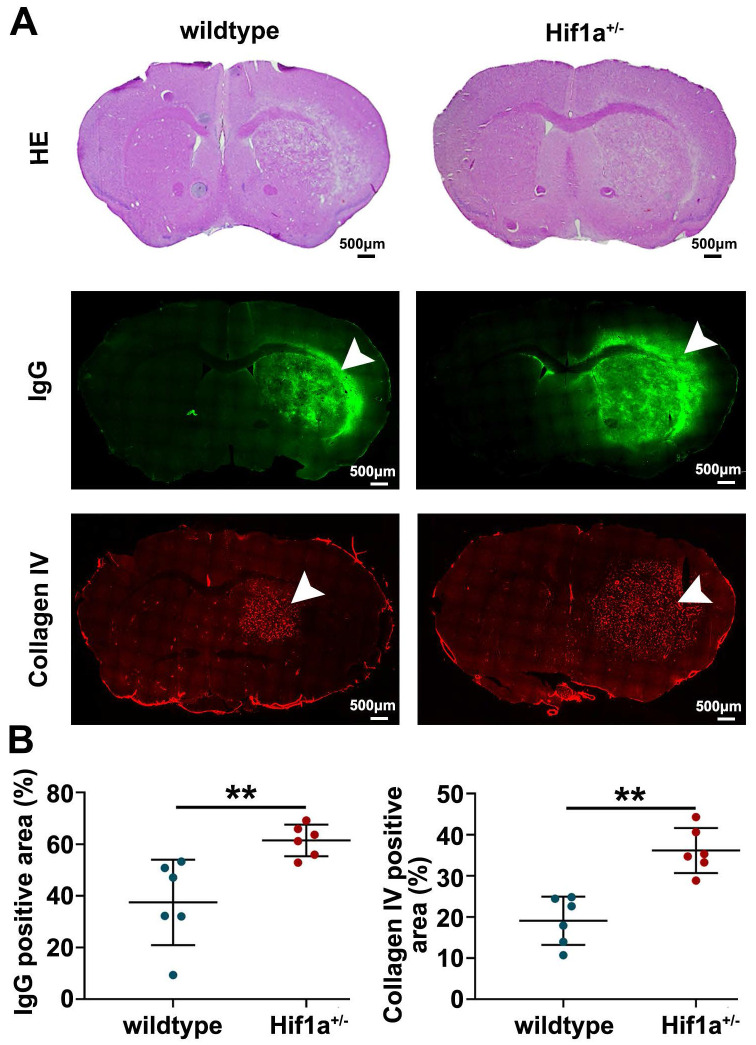
** Hif-1α gene deficiency exacerbates vascular damage after radiation**. (A) Representative H&E staining, IgG (a marker of vascular disruption), and Collagen IV (a marker of blood vessel) immunostaining for wild-type and Hif-1α^+/-^ mice at 63 weeks post-fractionated 3×20 Gy irradiation. The scale bars are 500 μm. (B) Quantification of the percentage of IgG positive staining area and Collagen IV positive staining area in ipsilateral hemisphere area, respectively. The Hif-1α^+/-^ mice exhibited higher levels of IgG leakage and more malformation of blood vessels in the brain tissue. Student t-test. N=6 brain slices in each group. ^**^P<0.01.

Furthermore, the Hif-1α^+/-^ mice exhibited higher levels of IgG leakage, which is considered one of the markers of vascular disruption (Figure 5). Additionally, we observed more malformation of blood vessels, as characterized by increased vessel diameter in the brain tissue of Hif-1α^+/-^ mice (Figure 5). These results indicate that Hif-1α gene deficiency exacerbates vascular damage after radiation.

## Discussion

Glioblastoma is characterized by infiltrative growth, intracerebral metastases, and resistance to chemotherapy, complete eradication after standard treatment regimens (surgical resection followed by radiotherapy plus concomitant and adjuvant temozolomide) is extremely difficult 13-15. Unfortunately, patients often face a high rate of recurrence and dismal prognosis. While increasing the radiotherapy dose may improve the success rate, it inevitably leads to brain damage. Actively dividing cells, including oligodendrocytes, vascular endothelium, and various precursor cells, are particularly vulnerable to this damage. Therefore, severe side effects preclude higher doses of radiotherapy 16. This gloomy reality has dominated for decades, but there is hope for a long-awaited breakthrough, thanks to new developments in regenerative medicine. While cell replacement strategies for brain repair have brought mixed results and have yet to prove successful in replacing neurons, replacing glial cells 17, 18 or vascular components 19 is highly feasible. It creates an opportunity for innovation in treating GBM by escalating the radiotherapy dose followed by restorative therapy.

Our initial studies have shown that elevating the single dose of radiotherapy to as high as 80 Gy in mice is well tolerated over several weeks (data not shown), which is encouraging as this time window would allow regenerative intervention. However, despite an initial tumor response, the tumor could not be entirely eradicated, and recurrence was observed within two weeks. Therefore, we explored the effect of ultra-high dose fractionated radiation for brain tumor eradication and found that a fractionated 3×20 Gy radiation dose can fully eradicate the tumor and significantly extend the survival time of mice while only resulting in mild brain injury over 12 weeks.

A variety of glial cells are extremely vulnerable to irradiation. These include astrocytes and microglia 20, but, most importantly, the oligodendrocytes and oligodendrocyte progenitor cells 21. Oligodendrocytes are post-mitotic cells that provide a myelin sheath for neurons, and it has been shown that oligodendrocytes undergo continuous turnover and are replaced by local tissue progenitors 22, 23. Depletion of these cells has been reported to occur as early as 3 days after irradiation 24, ultimately resulting in demyelination and white matter loss, which are features typically associated with post-irradiation brain atrophy. Astrocytes are another important glial phenotype to consider. Given their recently elevated functionality, including maintaining potassium and neurotransmitter homeostasis, calcium signaling, and the production of cytokines, as well as playing a critical role in the formation of the BBB 25, it is warranted to assess the contribution of astrocytes to radiation injury, and consider them as a therapeutic target.

The vascular consequences of radiotherapy have been relatively well characterized and include early and chronic effects in the brain 26, 27. Early effects manifest as denudating endothelial cells, parenchymal cell damage, and exhaustion of the cell renewal system. It is followed by abnormal proliferation of the endothelial cells, which are critical components of vascular remodeling, occurring months after the initial exposure. It has been shown that clinically relevant doses of ionizing radiation cause changes in the permeability of the BBB due to the abnormal synthesis of multiple proteins 28. Vascular changes tend to predominate and can range from thrombosis, hemorrhage, and hyalinization to fibrinoid necrosis, which can further exacerbate the hypoxic/ischemic necrosis in the area. Critical to the feasibility of our study is the slow development of vascular abnormalities, which, while preceding necrosis, still require a long time to evolve 27.

Inadequate repair of damaged endothelial cells and BBB disruption after radiation exposure increase hypoxia in the local microenvironment, leading to upregulation of hypoxia-inducible factors. Hif-1α and Hif-2α are master transcription factors for the cellular response to hypoxia 29. Inhibition of Hif-1 activity is believed to have therapeutic benefits in tumor treatment 30. It has been shown that oligodendrocytes and oligodendrocyte precursor cells (OPCs) play a central role in cerebral angiogenesis* via* Hif signaling. Yuen *et al.* found that constitutive Hif-1/2α stabilization led to OPC maturation arrest, induced excessive postnatal angiogenesis *in vivo*, and directly stimulated (in a paracrine mechanism) endothelial cell proliferation *in vitro*. Conversely, OPC-specific Hif-1/2α loss of function led to insufficient angiogenesis in the corpus callosum and extensive axonal loss 31. These findings point to critical interactions between OPCs, angiogenesis, and axonal integrity, with Hif signaling being a master regulator. Dysregulation of this system during radiation therapy may be the focal point in the pathomechanism of radiation-induced brain injury. Indeed, this is highly feasible, as the early elimination of OPCs after radiotherapy removes their pro-angiogenic role and leads to the deterioration of endothelial cells. The exact mechanism could also contribute to the failed recruitment of new pro-angiogenic precursors despite their presence in circulating blood.

Regenerative strategies offer the potential to alleviate or even eliminate the side effects of anti-tumor treatment 18. An important implication is an opportunity to elevate radiation doses beyond the currently acceptable maximum dose. There is a precedent for this approach in hematology, where treating malignancies involves delivering a high dose of chemo/radiotherapy to the whole body, thereby completely destroying the tumor cells and hematopoietic stem cells; however, these stem cells can be restored *via* bone marrow transplantation 32. This method is now a standard of care, with spectacular outcomes and, frequently, a complete cure for some malignancies 33. Similarly, repairing or replacing damaged glial and vascular components after radiation injury seems feasible. Highly potent neural progenitor populations, such as glial-restricted progenitors and human neural progenitor cells transduced with GDNF, have been shown to replace endogenous dysfunctional glia in myelin disease models or amyotrophic lateral sclerosis 34, 35. Moreover, the feasibility of an oligodendrocyte replacement strategy after low-dose radiotherapy has been demonstrated but without tumor eradication 18. In our study, we take this approach a step further by using a higher radiation dose (60 Gy delivered as three daily fractions of 20 Gy each) to completely eliminate the tumor and investigate the possibility of preventing late-stage white matter and vascular damage through a regenerative strategy.

Our study presents several novel contributions that distinguish it from previous research: 1) Rationale for dose escalation: We provide a compelling rationale for escalating the radiation dose in large brain volumes, addressing the challenges encountered with conventional approaches. While previous studies have explored ultra-high dose rate radiotherapy (FLASH) for glioblastoma, these techniques solely target the bulk of the tumor, leaving satellite cancer cells dispersed within the brain, leading to a high recurrence rate. In contrast, our study investigates the efficacy of an ultra-high fractionated 3×20 Gy radiation dose applied beyond tumor margin, demonstrating its potential to fully eradicate the tumor and significantly prolong the survival time of mice. Remarkably, this treatment regimen resulted in only mild brain injury over a three-month period, providing a broad window of time devoid of neurological deficits and featuring a delayed onset of vascular damage happening before neuronal loss. This extended timeframe offers a unique opportunity to intervene by enhancing the function of brain vasculature, with Hif1α identified as one potential pathway for targeted intervention. 2) We utilized immunocompetent mice for tumor inoculation and radiotherapy studies, which provides a more clinically relevant model for investigating brain tumor-host interactions and testing therapeutic strategies. Most human tumors are established in immunodeficient recipient mice (nude*/rag2/scid*) to prevent xenograft rejection, but this approach lacks an adaptive immune system, limiting its relevance for studying anti-tumor treatment. In contrast, our use of C57BL/6J immunocompetent mice under a tolerance induction regimen allowed us to investigate the effects of aggressive radiotherapy for this devastating disease in a more clinically relevant brain tumor model. 3) Assessment of Hif signaling under radiation: Hif signaling is a well-established system that proved to be a master regulator of endothelial homeostasis and angiogenesis. This signaling, however, has not been systematically studied in the context of early responses to radiation injury, and the modulation of Hif signaling has not been exploited as a therapeutic strategy for radiation injury of the brain. Here, we evaluated the status of Hif signaling after ultra-high dose radiotherapy and showed that Hif-1α deficiency aggravated local vascular damage after radiation. This important result provides initial evidence for developing a strategy to prevent radiation injury *via* intervening with Hif-1α signaling.

Overall, we propose a completely new approach for treating brain tumors with the perspective of using regenerative medicine methods. We have demonstrated that supportive cellular components in the brain (glia and vasculature) are radio-sensitive but also have the potential to be replaced, which could open entirely new treatment paradigms for neoplastic conditions in the brain. This approach could be applied to chemotherapy, where targeted intraarterial delivery techniques are now available but cannot be fully exploited due to excessive toxicity. Besides this, our findings also shed light on the consequences and therapeutic targets of extremely high doses of radiation, which could be relevant in the context of nuclear accidents or catastrophes. Collectively, our study offers new insights into the use of regenerative medicine in treating brain tumors and provides a foundation for future research in this area.

## Supplementary Material

Supplementary figures.

## Abbreviations

AV: average
BBB: blood-brain barrier
BLI: bioluminescence imaging
CT: computed tomography
FLASH: ultra-high dose rate in radiotherapy
GBM: glioblastoma
H&E: hematoxylin and eosin
Hif-1α: hypoxia-inducible factor- 1 alpha
MRI: magnetic resonance imaging
OPCs: oligodendrocyte precursor cells
ROIs: regions of interest
TE: echo time
TR: repetition time
