# Supplementary material for: Unlocking the Potential of Ultra-High Dose Fractionated Radiation for Effective Treatment of Glioblastoma in Mice
Source: J Cancer. 2024 May 30;15(13):4060–71. doi: 10.7150/jca.95148 (PMC11212101; doi:10.7150/jca.95148)
Supplement: Supplementary file 1 — Supplementary figures. [file jcav15p4060s1.pdf]

Unlocking the Potential of Ultra-High Dose Fractionated Radiation for Effective Treatment of Glioblastoma in Mice

Xiao-Yan Lan[1,2*], Lukasz Kalkowski[2*], Cheng-Yan Chu[2], Anna Jablonska[2], Shen Li[3], Mihoko Kai[4], Yue Gao[2], Miroslaw Janowski[2], Piotr Walczak[2]

[1]Department of Neurology, Dalian Municipal Central Hospital, Dalian, China

[2]Department of Diagnostic Radiology and Nuclear Medicine, University of Maryland Baltimore, Baltimore, MD, USA

[3]Department of Neurology and Psychiatry, Beijing Shijitan Hospital, Capital Medical University, Beijing, China

[4]Department of Radiation Oncology, Johns Hopkins University, School of Medicine, Baltimore, MD, USA

*These authors contributed equally to this work.

Correspondence to: Prof. Piotr Walczak, MD, PhD, Department of Diagnostic Radiology and Nuclear Medicine, University of Maryland Baltimore, 670 W. Baltimore St., Baltimore, MD 21201, Phone: +1(410)7067904, Email: pwalczak@som.umaryland.edu.

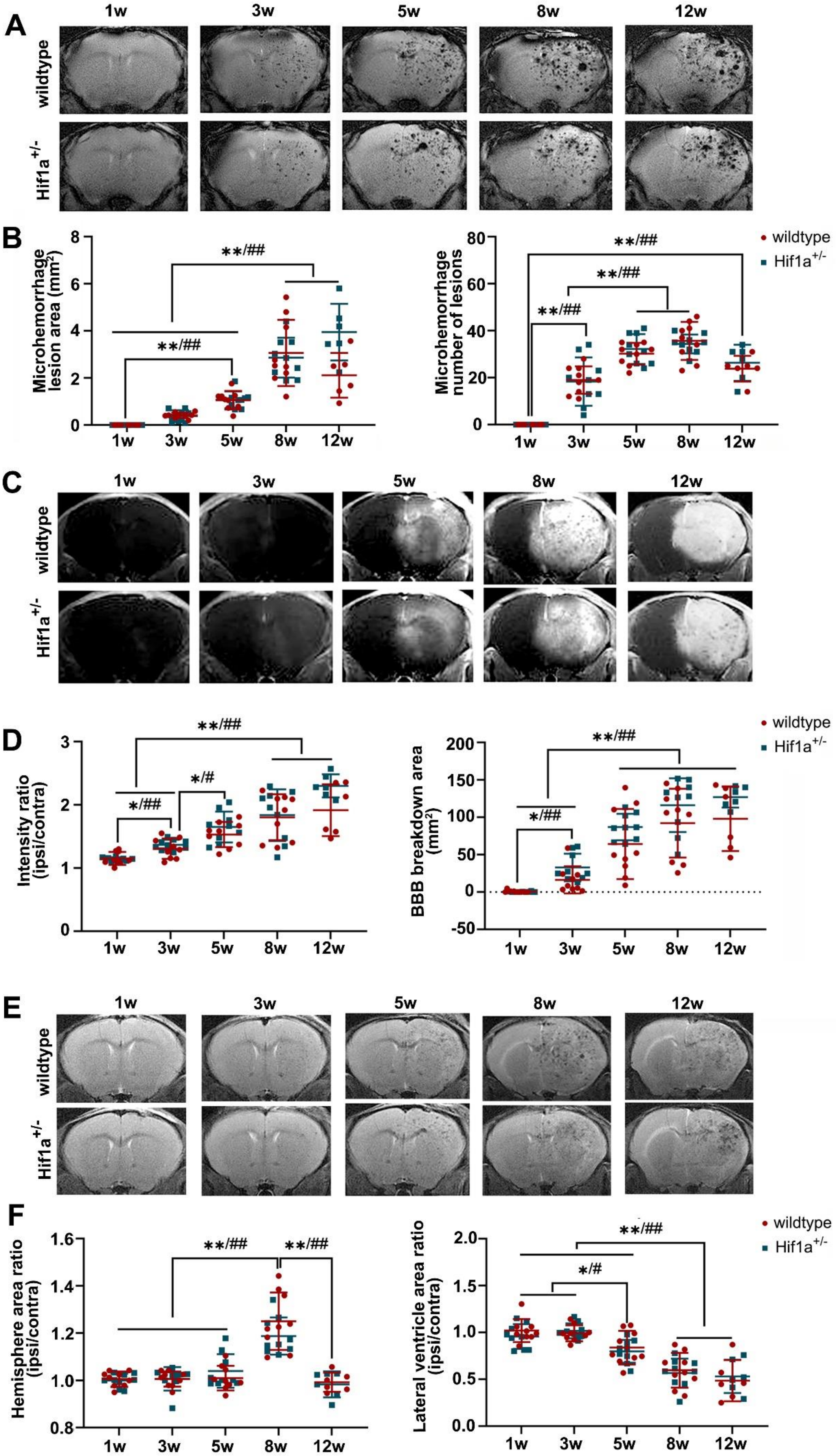
A
1w
3w
5w
8w
12w
wildtype
Hif1a+/-
B
Microhemorrhage lesion area (mm2)
**/##
wildtype
Hif1a+/-
Microhemorrhage number of lesions
C
D
Intensity ratio (ipsi/contra)
*/##
*/#
BBB breakdown area (mm2)
E
F
Hemisphere area ratio (ipsi/contra)
Lateral ventricle area ratio (ipsi/contra)

Supplementary Figure 1. Ultra-high fractionated 5×20 Gy radiation-induced brain injury in Hif-1α$^{+/-}$ heterozygote and wildtype mice. (A-B) Representative T2* images and microhemorrhage assessment for mice receiving fractionated radiation. (C-D) Representative T1-weighted gadolinium-enhanced MR images and BBB status for mice receiving fractionated radiation. (E-F) Representative T2-weighted MR images and anatomical analysis for mice receiving fractionated radiation. N=3 mice per group (3 slices for each mouse). Wildtype group: $^{*/**}P < 0.05/0.01$. Hif-1α$^{+/-}$ group: $^{\#/\#\#}P < 0.05/0.01$.

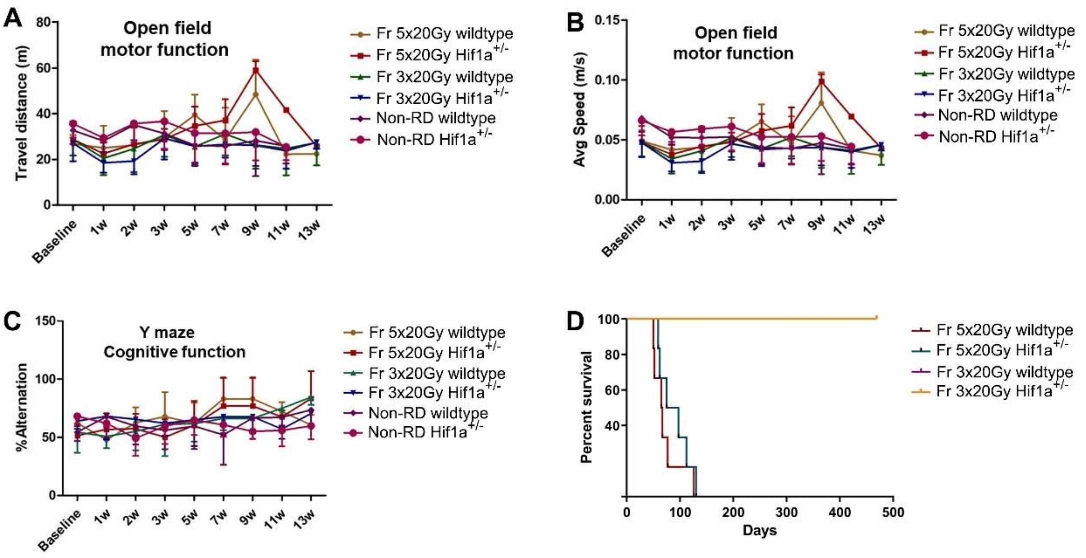

Supplementary Figure 2. Behavioral tests and animal survival time of Hif-1α$^{+/-}$ and wildtype mice after fractionated 5×20 Gy or 3×20 Gy radiation. (A-B) An open field test indicated no motor function difference between wildtype and Hif-1α$^{+/-}$ mice. (C) Y maze test showed no cognitive function difference between wildtype and Hif-1α$^{+/-}$ mice. (D) Survival curves showed no difference between Hif-1α$^{+/-}$ heterozygote mice and wild-type mice under any radiation dose. N=6 mice/group.
